# Photocurrent generation in carbon nanotube/cubic-phase HfO_2_ nanoparticle hybrid nanocomposites

**DOI:** 10.3762/bjnano.7.101

**Published:** 2016-07-26

**Authors:** Protima Rauwel, Augustinas Galeckas, Martin Salumaa, Frédérique Ducroquet, Erwan Rauwel

**Affiliations:** 1 Institute of Physics, University of Tartu, Ravila 14c, 51014 Tartu, Estonia; 2 Department of Physics, University of Oslo. P.O. Box 1048 Blindern, 0316 Oslo, Norway; 3 Tartu College, Tallinn University of Technology, Puiestee 78, 51008 Tartu, Estonia,; 4 IMEP-LAHC, CNRS, Université de Grenoble-Alpes, Minatec campus, 38016 Grenoble, France

**Keywords:** carbon nanotubes, HfO_2_, nanoparticles, photocurrent

## Abstract

A hybrid material consisting of nonfunctionalized multiwall carbon nanotubes (MWCNTs) and cubic-phase HfO_2_ nanoparticles (NPs) with an average diameter of 2.6 nm has been synthesized. Free standing HfO_2_ NPs present unusual optical properties and a strong photoluminescence emission in the visible region, originating from surface defects. Transmission electron microscopy studies show that these NPs decorate the MWCNTs on topological defect sites. The electronic structure of the C K-edge in the nanocomposites was probed by electron energy loss spectroscopy, highlighting the key role of the MWCNT growth defects in anchoring HfO_2_ NPs. A combined optical emission and absorption spectroscopy approach illustrated that, in contrast to HfO_2_ NPs, the metallic MWCNTs do not emit light but instead expose their discrete electronic structure in the absorption spectra. The hybrid material manifests characteristic absorption features with a gradual merger of the MWCNT π-plasmon resonance band with the intrinsic defect band and fundamental edge of HfO_2_. The photoluminescence of the nanocomposites indicates features attributed to combined effects of charge desaturation of HfO_2_ surface states and charge transfer to the MWCNTs with an overall reduction of radiative recombination. Finally, photocurrent generation under UV–vis illumination suggests that a HfO_2_ NP/MWCNT hybrid system can be used as a flexible nanodevice for light harvesting applications.

## Introduction

Nanoparticles (NPs) have emerged as promising candidates for many applications due to their unique electronic, optical and magnetic properties compared to their bulk counterparts. During the last decade, composite materials have spurred large interest, and with the rise of nanotechnology, the development of new nanocomposite materials promoting new properties has taken a step forward. These nanocomposite materials will be the key components for the development of new applications in the field of nanotechnology. It is well known that by combining different classes of materials, one can obtain nanocomposites exhibiting properties of the individual materials along with new characteristics as a result of hybridization. For instance, the combination of nanoparticles with carbon nanotubes (CNTs) has proven to greatly broaden the area of potential applications, such as gas sensors [1–2], solar cells [3–4], bioimaging [5] and IR detectors [6], most of which require efficient charge transfer from the nanoparticle to the CNT and charge conduction via the CNT. To date, numerous studies have been reported on the decoration of CNTs with metal oxides including TiO_2_ [7–8] and ZnO [9] for solar cell applications and SnO_2_ for gas sensors. Reports on the fabrication of an all carbon nanocomposite combining CNTs, graphene and carbon quantum dots (CQDs) are available [10]. Recently, Yu et. al studied the charge transfer mechanism in CQD–graphene composite and have emphasized its potential as a hot carrier, solar cell material [11]. However, CQDs still need further investigation as their optical properties tend to vary with the synthesis route, their size and the functional groups surrounding them [12].

HfO_2_ compounds and their solid solutions have been recognized as important materials in the development of technology [13], and more particularly, in the field of transistor technology [14]. In fact, HfO_2_ is a dielectric material with a band gap of 5.7 eV [14–15]. HfO_2_ has already been integrated in numerous technologies and has been chosen for the replacement of Si-based gate oxides in advanced complementary metal-oxide semiconductors (CMOS) [16]. Many efforts have been made towards the stabilization of the cubic phase of HfO_2_ due to its more interesting properties such as higher dielectric permittivity via doping [17] and substrate-induced strain. Many systems and processes were developed to reach this goal. One advantage of studying this material for other properties is that the microelectronic industry already produces and employs HfO_2_-based devices and can therefore facilitate its integration into nanosystems. This further expedites the development and integration of new technologies based on HfO_2_ owing to already existing technological platforms. Major efforts are being directed worldwide towards mastering the dimensionality of nanoparticles; among established methods ensuring control of both size and shape is the nonaqueous sol–gel route [18–19]. In our previous studies, a strong photoluminescence in the visible range was observed from HfO_2_ nanoparticles under below-bandgap excitation [20–21] and attributed to surface-located intrinsic and extrinsic defects arising from Hf^3+^ and O^2−^ vacancies. The small diameter of the nanoparticles in the present study, 2.6 nm on average, implies a very high surface-to-volume ratio, and consequently, enhanced surface-defect-related luminescence. Furthermore, for the cubic-phase HfO_2_ nanoparticles, oxygen vacancies acting as luminescence trap states are present in large amounts [22]. In the variety of different techniques used to decorate CNTs, the first step is usually the dispersion of the CNTs in a liquid solution as they exist in the form of bundled ropes [23]. Acid treatment of the CNTs is typically the preferred method offering a two-fold advantage; not only does it debundle the CNTs, but it also creates functionalized groups on the side walls which, in turn, prevent the CNTs from rebundling. In addition, these functional groups also act as anchor sites for nanoparticles in the process of CNT decoration. In fact, acid treatment creates defects (vacancies and holes in the side walls) on the CNT surfaces along with carboxyl groups in the case of carboxylic acid treatment [24]. The bond with these carboxyl groups is then created via hydroxyl groups present on the surface of the nanoparticles on forming esters [25]. Moreover, π–π stacking has also proven effective in attaching inorganic metal oxide nanoparticles to the surface of nonfunctionalized nanotubes. In this approach, the aromatic ring of the CNT is directly connected to the benzyl ring of the inorganic nanoparticles [26–27]. Birojou et al. have also observed that in the case of nonfunctionalized graphene decorated by gold nanoparticles via electrostatic interactions, the defect sites on the graphene are preferentially decorated by the Au nanoparticles with an increase in the sp^2^ hybridization of graphene in these regions [28]. In another approach, chemical functionalization is usually combined with ultrasonication. In fact, the latter tends to create defects on the walls of the CNT along with C dangling bonds along with a change in the carbon hybridization from sp^2^ to sp^3^ [29]. Nevertheless, studies have been conducted where ultrasonication without functionalizing agents has been used for successfully debundling CNTs with minimal damage to the tube walls [23].

CNTs produced via CVD methods typically contain various imperfections, such as residual impurities of metal catalysts, graphene sheets, amorphous carbon and different defects [30–31], generally qualified as either topological or localized. The most common defects are comprised of edges and dangling bonds in addition to pentagonal and heptagonal defects, creating bends in the CNTs known to accommodate foreign atoms in the sp^2^-hybridized carbon matrix [32–33]. Some defects, including five-membered rings responsible for closing the ends of nanotubes, are also present at bends, Y-junctions and kinks in nanotubes. In general, these defect sites are highly reactive and provide anchor sites to fix nanoparticles whose surfaces have organic moieties [34]. Furthermore, certain defects also produce nanotube curvature, which in turn creates π-orbital mismatch and consequently creates more active sites on the CNT. In any case, the presence of these defects is known to affect the band structure of the carbon nanotubes and thus can be studied by means of optical absorption and photoluminescence spectroscopy [25].

We have already reported elsewhere that the free-standing cubic HfO_2_ nanoparticles are luminescent on their own with characteristic emission in the blue-green region of the visible spectra. This may be attributed to surface defects including Hf and O vacancies [21]. The motivation of the present work is different from other well-studied luminescent nanocomposites containing TiO_2_ and ZnO that are investigated around band gap excitation. Here, contrary to the previous two semiconductors, novel optical properties and photocurrent generation are expected from the hybrid material studied in under band gap excitation conditions. Nevertheless, one has to also consider that nanoparticles in contact with the CNT may undergo surface passivation along with corresponding changes in the electron trap states within the band gap [35]. Furthermore, since the CNTs are not functionalized in the present study, a direct insight into the role of intrinsic defects in the as-grown CNTs can be attained in the process of attaching nanoparticles to their surfaces. Similarly, the absence of functional groups on the CNT provides a direct electrical contact between the CNT and HfO_2_ NP along with a better understanding of the individual contribution of CNTs to the photoluminescence properties and photocurrent generation in the hybrid material.

## Results and Discussion

### Structure and morphology

The overall morphology of the hybrid material was studied by TEM. The various HAADF-STEM images presented here give an overview of the morphology of the hybrid nanomaterial. Most of the HfO_2_ nanoparticles are agglomerated and appear to be attached only in certain particular regions of the CNT. In our previous study we observed that cubic HfO_2_ nanoparticles on their own show a tendency to agglomerate [20]. The MWCNTs used in this study exhibited kinks, coils and buckling.

**Figure 1:**
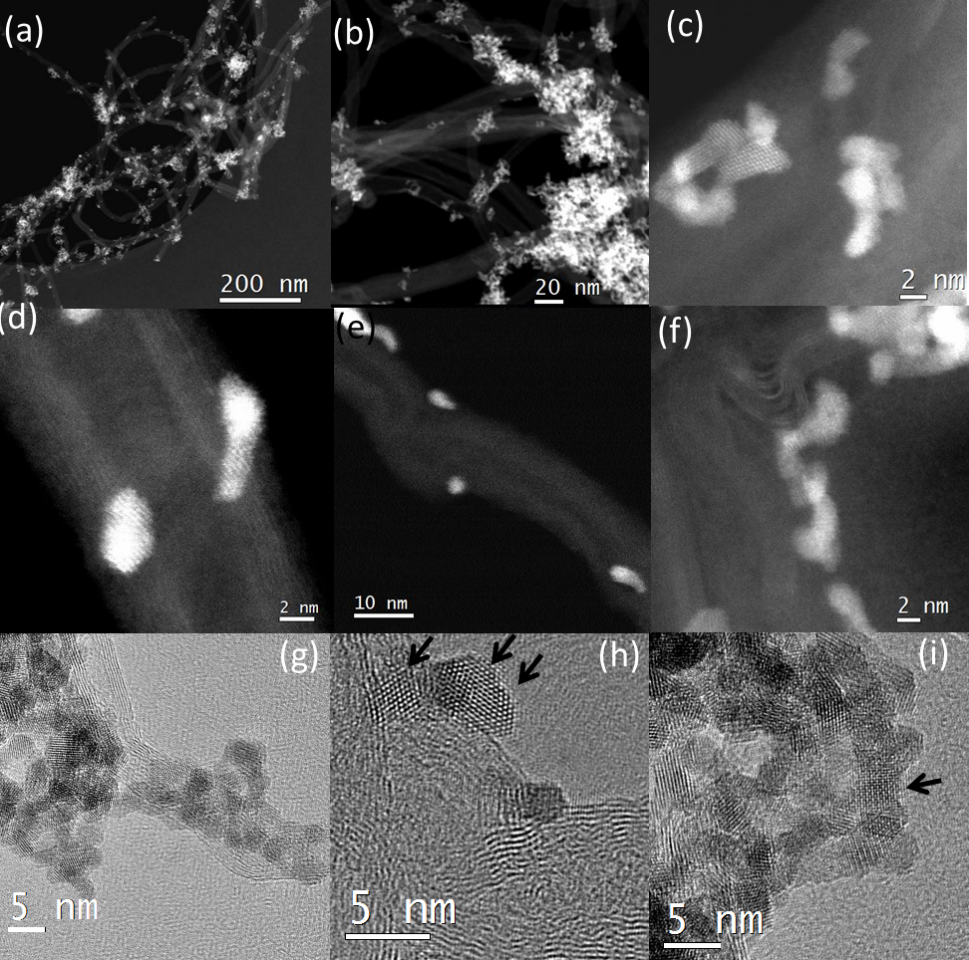
(a) HAADF-STEM image of the MWCNT:HfO_2_, (b) higher magnification image of (a), (c) HAADF-HRSTEM images of the HfO_2_ NPs on the MWCNTs, (d) NPs attached to the side walls of a MWCNT, (e) NPs attached at buckled edges of the MWCNT, (f) wall of the nanotube showing defects and irregularities to which nanoparticles are attached, (g) HRTEM images of randomly oriented agglomerates, (h) <110>-oriented, and (i) <001>-oriented nanoparticles indicated by arrows and all attached to curved regions of the CNT.

In Figure 1a, an overview of the hybrid material shows successful attachment of the nanoparticles to the CNT. The cubic HfO_2_ nanoparticles exhibit an average diameter of 2.6 nm [20]. In the overview of Figure 1a, the nanoparticles seem to decorate only specific sites either singularly or in agglomerates. A higher magnification HAADF-STEM image in Figure 1b further reinforces the selective decoration of the HfO_2_ nanoparticles as several regions of the CNT are devoid of nanoparticles. An explanation to this selective anchoring is provided below and also in the EELS section. In Figure 1c, a HAADF-HRSTEM image exhibits small agglomerates of these nanoparticles on the MWCNT, where the diameter of the MWCNT has reduced due to buckling. The nanoparticles remain crystalline as displayed by the atomic column resolution, even after 2 h of sonication. In Figure 1e, a defective nanotube presenting successive kinks is shown with the HfO_2_ nanoparticles preferentially attached around the kinked area. A HRSTEM image in one such buckled/kinked regions of the MWCNT is illustrated in Figure 1f, where the nanoparticles are attached to two different kinked areas. In effect, in the kinked region, there is a breakdown in the curvature and CNTs with individual walls exhibit different curvatures. Moreover, when the MWCNT is bent, the changes in the curvature induce a modified atomic arrangement and local break down of symmetry. This implies a local change of the electronic structure [36] along with an increase in π mismatch. This in turn accentuates the reactivity of these MWCNTs and converts these defects into receptors for functional groups on the surface of the nanoparticles. Buckling and kinking of the MWCNT arises due to rearrangement of C atoms around the curved area giving rise to vacancies and dangling bonds. These defective areas are well known for their high reactivity to foreign atoms [37].

In Figure 1d, nanoparticles are attached to the side walls of the MWCNT. It has to be emphasized here that according to literature reports, attachment of nanoparticles to the sidewalls of MWCNTs appears to be only possible via functionalization of the sidewalls. In our case, no functionalization has taken place, nor in the image do we observe the presence of curved regions or topological defects that facilitate the decoration of these nanoparticles to the MWCNT. This mechanism of anchoring to the sidewalls is further probed in the EELS section. On the other hand, the side walls of the MWCNT do not appear to be atomically flat, and the outermost walls are not distinct, indicating damage to the walls of the MWCNT which is a result of the sonication treatment and will be discussed in a forthcoming paper. This provokes a breakdown in the graphitic structure, which once again affects the electronic structure and increases the reactivity of the MWCNT to foreign atoms.

### Optical properties

**Figure 2:**
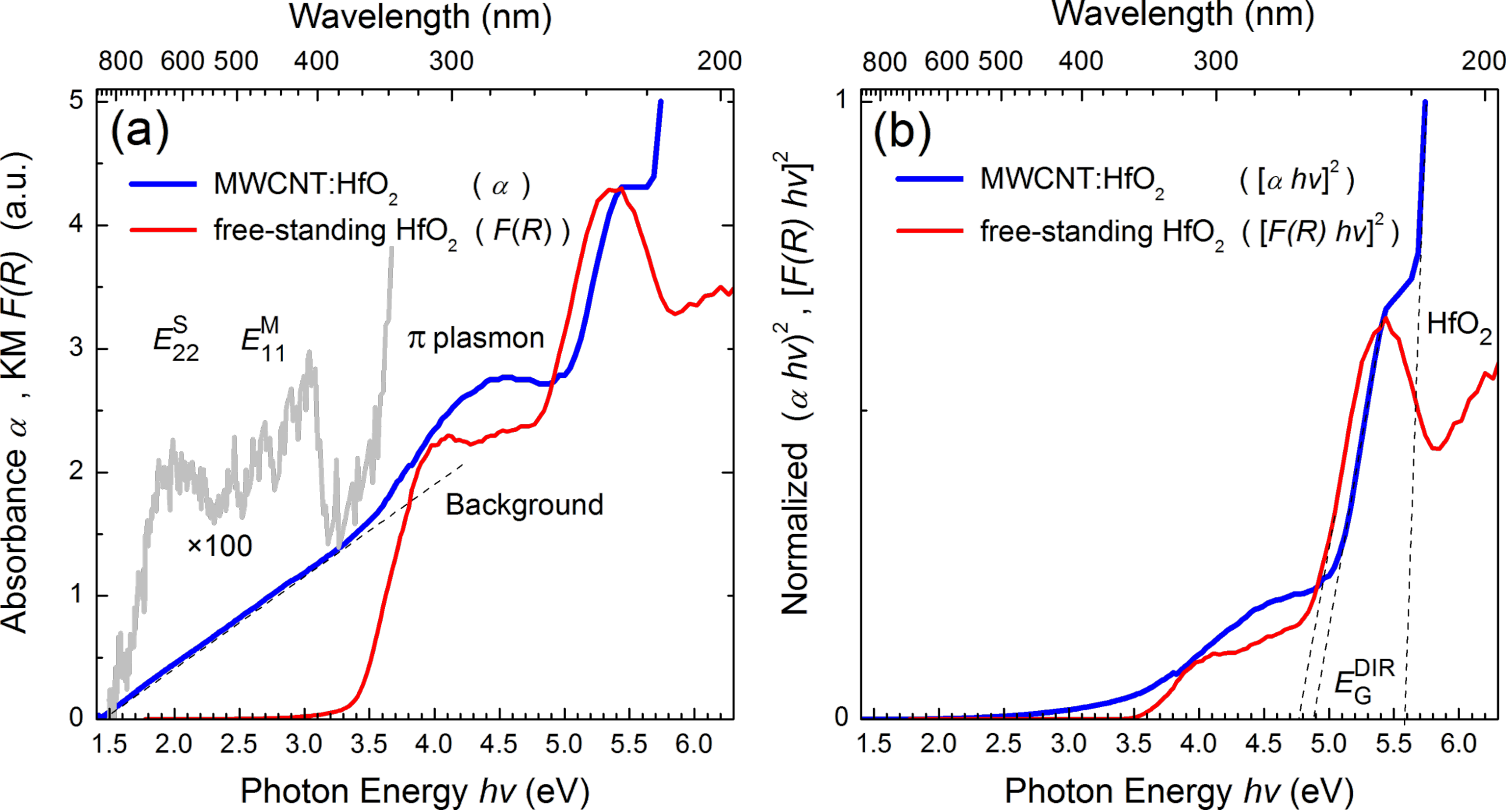
(a) Absorption spectrum of MWCNTs decorated with cubic HfO_2_ nanoparticles obtained from the transmittance measurements of colloidal suspension in ethanol (blue curve) against that of free-standing HfO_2_ nanoparticles from the diffuse reflectance measurements (red curve). (b) Tauc plots of the corresponding dependencies, absorbance (α) and Kubelka–Munk function (KM *F*(*R*)), representing the case of direct optical transitions.

The distinctive optical properties of CNTs derive from electronic transitions within the one-dimensional density of states (DOS), which is discontinuous in nature and exhibits sharp peaks called van Hove singularities (vHS) [38]. The energy separations between the vHS in the valence and conduction bands depend on the nanotube structure, thus optical absorption and emission spectroscopy allow identification of the CNT chirality and diameter as well as quality in terms of nontubular carbon content and structural defects. The characterization of multiwalled CNTs, however, is challenging because of the involvement of several shells with different structure and typically higher defect concentration compared to single-walled CNTs. The optical absorption properties of the hybrid MWCNT:HfO_2_ nanocomposites deduced from the room temperature transmittance measurements of a colloidal suspension in ethanol are summarized in Figure 2.

The fine structure of the absorption spectrum in the UV–vis region revealed upon background subtraction and ×100-fold magnification is represented by the grey curve in Figure 2a. One can observe an apparent double feature in the absorption spectrum in the photon energy region 1.5–3.5 eV, where typically the vHS-related bandgap transitions are observed in CNTs, such as *E*_22_ from the semiconducting SWCNT and *E*_11_ from the metallic SWCNT [39]. At higher photon energies, a broad absorption band emerging at around 4.5 eV is likely due to the π-plasmon resonance [40–41]. This feature gradually merges with the fundamental absorption edge of HfO_2_ building up at around 5.6 eV. These assertions are supported by the similarity of the absorption edge and absence of CNT-related features in the spectrum of free-standing cubic HfO_2_ nanoparticles obtained earlier from the diffuse reflectance measurements [21] and put alongside for comparison in Figure 2. The absorption thresholds marked by the dashed lines in the Tauc plot in Figure 2b are consistent with the reported bandgap energies for hafnia (theoretical 4.9–5.7 eV and experimental 5–6 eV [14,42]) as well as with defect-related absorption showing up close to the fundamental edge and commonly associated with oxygen vacancies [43–44]. With regard to the latter, a noteworthy discrepancy (at ≈0.1 eV) of the absorption thresholds in the case of CNT-embedded and free-standing HfO_2_ nanoparticles likely points towards different charge states of oxygen vacancies or differently coordinated vacancies in the same charge state. Both scenarios seem feasible bearing in mind that the comparison involves suspended colloidal versus free-standing particles and that the relevant defects are located predominantly near or on the surface. It is noteworthy in this respect that theoretical calculations of the electronic properties of oxygen vacancies in monoclinic HfO_2_ [45] predict single- and double-ionized vacancy states at 4.7 eV and 4.9 eV above the valence band, which appear very close to the experimentally observed absorption thresholds in Figure 2b.

The electronic properties of CNTs are known to vary depending upon the chirality (wrapping angle) and diameter of the graphene sheet, generally exhibiting either semiconducting, or metallic behavior [46–48], the latter being observed in part of single-walled and in all multiwalled CNTs. In metallic CNTs, the electrons can be optically excited via a series of valence-to-conduction band transitions, resulting in characteristic vHS peaks in absorption spectra. However, since holes are instantly filled with readily available electrons, no excitons are formed, and consequently, no photoluminescence (PL) occurs in MWCNTs. Hence, PL measurements of our hybrid nanocomposites rather address mutual interaction of the radiative centers on the surface of HfO_2_ nanoparticles and metallic CNT framework, conceivably acting either as a surface-passivating agent or as an antenna enhancing light interaction with attached nanoparticles. Indeed, the nanotubes are known to induce optical quenching due to charge transfer from the nanoparticle to the CNT [49–51]. This charge transfer within conjugate species and CNTs usually occurs in the excited state [52]. In the reported case of CdSe/ZnS attached to CNTs, the optical quenching was attributed to a nonradiative energy transfer from the quantum dot to the SWCNT in the ground state [53].

**Figure 3:**
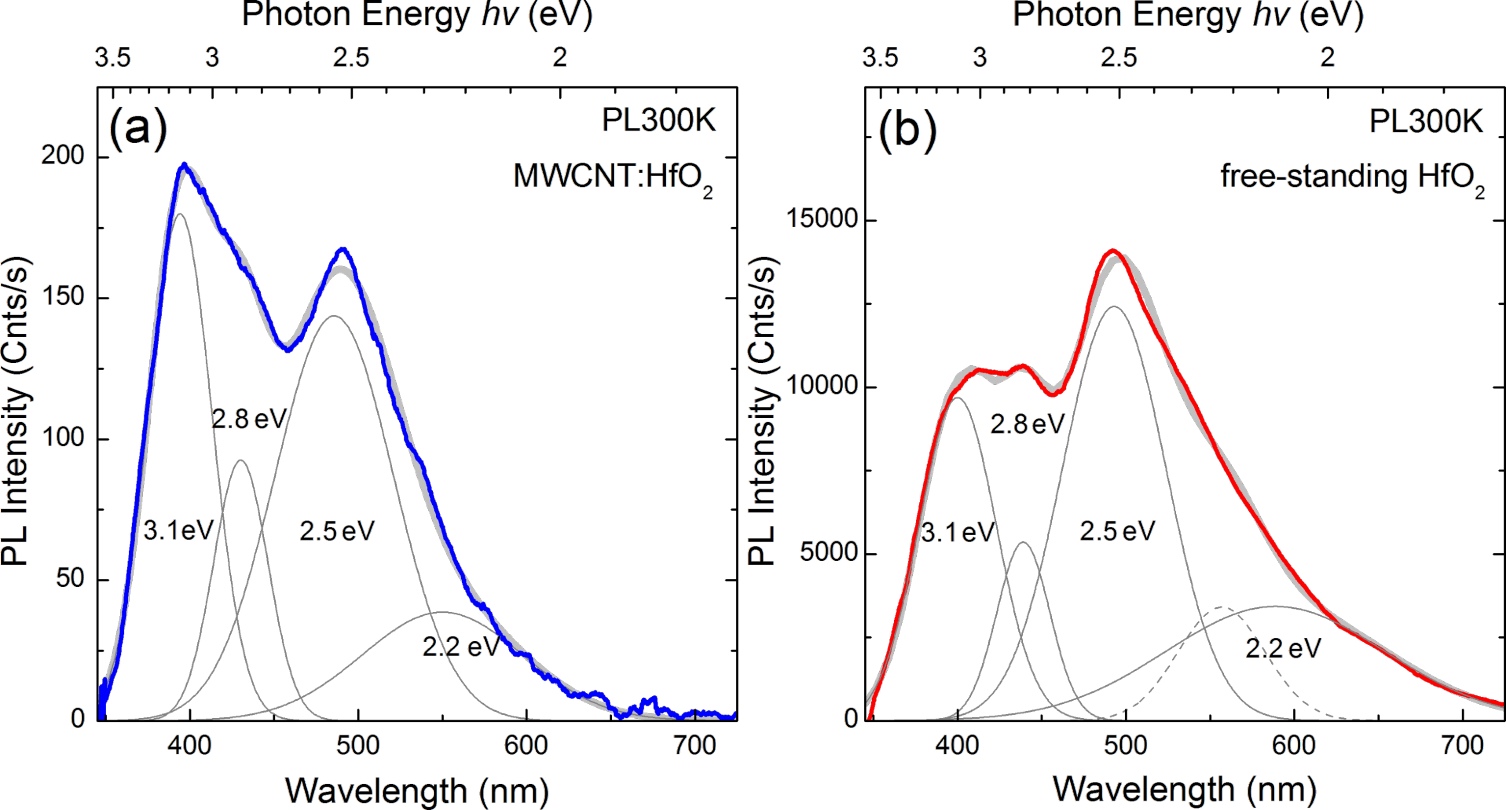
(a) Room temperature photoluminescence spectra of MWCNTs decorated with cubic HfO_2_ nanoparticles (from a colloidal suspension in ethanol) compared to (b) free-standing HfO_2_ nanoparticles. Grey curves represent the Gaussian deconvolution components. Note different the photoluminescence intensity scales in (a) and (b).

In the present study, we observe an overall decrease in the PL intensity when the HfO_2_ nanoparticles are attached to the CNT compared to the PL emission of the free-standing nanoparticles [21]. Figure 3 shows room temperature PL spectra of the hybrid nanocomposites dispersed in ethanol as a colloidal suspension and of the free-standing HfO_2_ nanoparticles. As one can deduce from the PL intensity scales in Figure 3a,b, the total quantum yield in the case of nanocomposites is two orders of magnitude lower compared to free-standing nanoparticles. In a first approximation, this could be attributed to the different densities of the photo-excited nanoparticles in both instances and also to charge transfer effects in CNTs. To gain some insight into the matter, the spectral content of the PL emission from nanocomposites and free-standing HfO_2_ nanoparticles was analyzed in more detail. As evident from Figure 3, the key constituents of the broad emission bands in both cases are represented by the same Gaussian deconvolution components centered at around 3.1, 2.8, 2.5 and 2.2 eV, albeit with a notable discrepancy in the relative strength of the 3.1 eV emission component. In the case of free-standing cubic HfO_2_ nanoparticles, the nature of strong visible emission combines surface defects that act as charge trapping centers and oxygen vacancies due to the large presence of Hf^3+^ in the structure. The photon energy range from 2 to 3 eV is identified in the literature as the typical response of the luminescent extrinsic centers associated with surface defects or impurities introduced during the synthesis of the nanoparticles. In particular, the characteristic green luminescence at around 2.5 eV is usually attributed to deep-level traps generated by oxygen vacancies, whereas the prominent emission component centered at 3.1 eV is related to Hf^3+^ defects on the surface of the nanoparticles. The attachment of the nanoparticles to a CNT not only provides a conducting pathway to evacuate the charges accumulated on the surface but also reduces the heating effects of the material due to nonradiative recombination.

### Core loss EELS C K-edge

**Figure 4:**
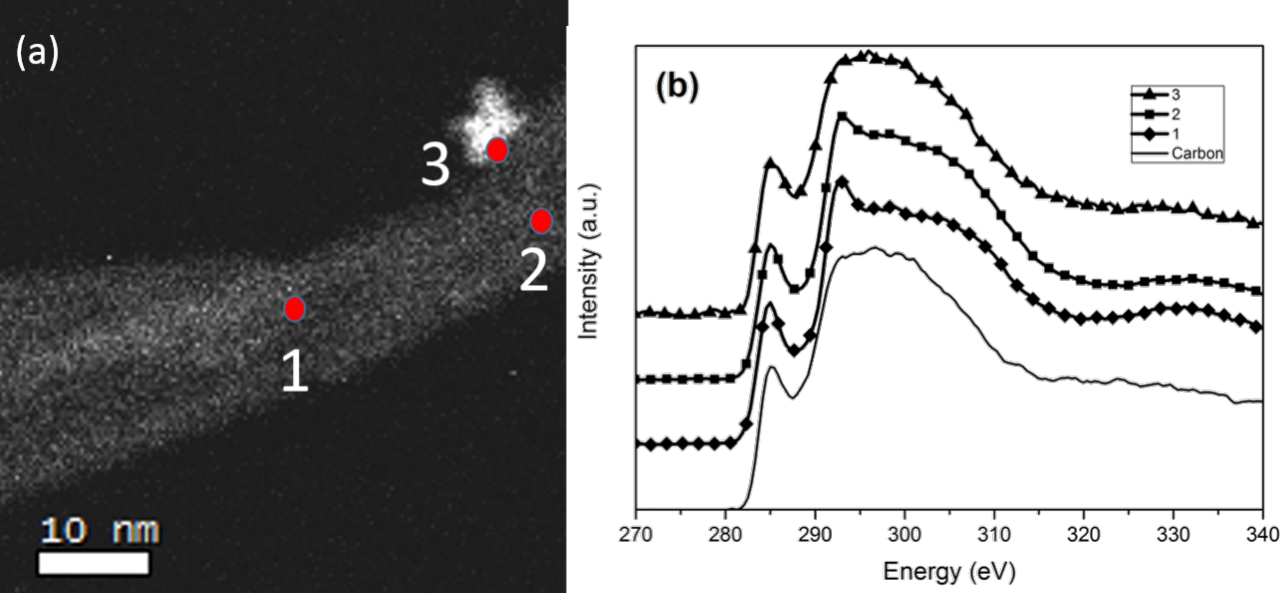
(a) HAADF-STEM image of the area of interest where EELS was performed, (b) C K-edge core loss EELS spectra on the area of interest indicated in Figure 2a.

Most nanoparticles are attached to sites with defects and changes in CNT curvature, creating π-orbital mismatches that increase the reactivity and allow nanoparticles with organic moieties to attach. However, there are regions on the CNT that have almost no curvature, as illustrated in Figure 1d. Since we have not functionalized the CNT, there are no functional groups created on the walls that facilitate NP anchorage. In such a case, it is relevant to gain insight into the nature of the graphitic layers in such areas of the CNT by probing their electronic structure with regards to sp hybridization. Energy loss near edge structure (ELNES) of the C K-edge was therefore used to probe the local electronic structure of the CNT at the nanoparticle interface in the noncurved regions of the CNT anchoring nanoparticles. In fact, several EELS spectra were taken from the hybrid nanocomposite at different places near the nanoparticles attached to the CNT. The EELS spectra were acquired from regions located far away from the carbon film of the TEM grid in order to exclude or minimize its influence on the C K-edge measurement. These areas of interest were at the holes of the holey carbon grid. Differences in the electronic structure along various points of the carbon nanotubes were probed in order to understand the affinity of these nanoparticles to only certain regions of the CNT. Accordingly, spectrum 1 in Figure 4b was acquired from point 1 in Figure 4a, which is devoid of nanoparticles, and indicates typical peaks for sp^2^/sp^3^ hybridization corresponding to multiwalled CNTs. The π* peak at 285 eV is related to the 1s to unoccupied antibonding π* transitions, and the exciton peak σ* at 292 eV arises due to transitions to the antibonding σ*. As we approach the region of the CNT decorated by nanoparticles (point 2, Figure 4a), we observe that the σ* peak in Figure 4b is smeared out along with a decrease in the intensity of the π* 285 eV edge. This smear is manifested as a broad hump starting above 288 eV and extending up to 305 eV, corresponding to the C 1s → σ* transition for disordered carbon–carbon bonds. On a similar note, other noticeable features are usually observed via X-ray absorption spectroscopy (XAS) in the region between π* and σ* transitions. These resonances are ascribed to the oxygen containing functional groups, i.e., a peak at 288.2 eV related to carbonyl (C=O) and another peak at 289.7 eV related to carboxylic (–COOH) [54]. In our case, we have not functionalized the nanotubes, therefore, the slight increase in the π*/σ* in ELNES C K-edge, arises due to the damage of the CNT walls [55], resulting in a loss of features in the σ* peak [54,56–58]. In the region of the CNT decorated by nanoparticles represented by point 3 in Figure 4a, we observe that the σ* peak in spectrum 3 of Figure 4b is even more smeared out with an increase in the intensity of the 285 eV edge and an even more featureless broad hump starting above 288 eV and extending up to 305 eV due to the C 1s → σ* transition, typical for extremely disordered carbon-carbon bonds. In fact, the smearing of this edge is characteristic of amorphous carbon, similar to the C-K edge of the amorphous carbon support of the TEM grid used as a reference, shown in Figure 4b.

Moreover, the apparent increase in the integrated area of the π* peak indicates an increase in the sp^2^ bonding fraction compared to other regions of the CNT and could be attributed to pyrolytic carbon as a result of sonication. Since a CNT is a rolled up sheet of graphite, defects in graphene include sp^2^ hybridized amorphous carbon harboring C–C defects with high reactivity, exploitable for the functionalization of graphene [59]. The breaking of the C–C bond is manifested as damage to the walls of the CNT and complete loss of medium to long range ordering [60]. This appears to be most conspicuous in places where the nanoparticles are attached to the CNT. Furthermore, amorphous carbon usually present in as-grown MWCNT contains a large number of dangling bonds [61] that also act as anchor sites via noncovalently bonded species. This further implies that C defects with dangling bonds act as anchor sites for nanoparticles whose surfaces are covered with functional groups, including amine groups. In our previous study, it was demonstrated that the HfO_2_ nanoparticles have amine-type capping layers around them. Furthermore, nitrogen species were observed from the XPS survey spectra performed on these free-standing HfO_2_ nanoparticles [20]. Thermo gravimetric analysis further confirmed that the surfaces of these nanoparticles are covered by organic species (14 wt %) that are mainly amine and reaction by-products typical of nonaqueous sol–gel routes using precursors that do not contain hydrate species. Here, the amine species capping acts not only as a surfactant but also as a shape and size controlling agent during growth of the NP. Since our MWCNTs were not acid treated, the particles are therefore not attached via a C–O–H bond. We can also exclude π–π interaction between the C ring of the CNT and the benzyl ring of probable benzylamine organic species on the surface of the nanoparticles. This implies that the most probable bonding mechanism is via a C dangling bond and N from the amine-type capping layer.

### Photocurrent response

**Figure 5:**
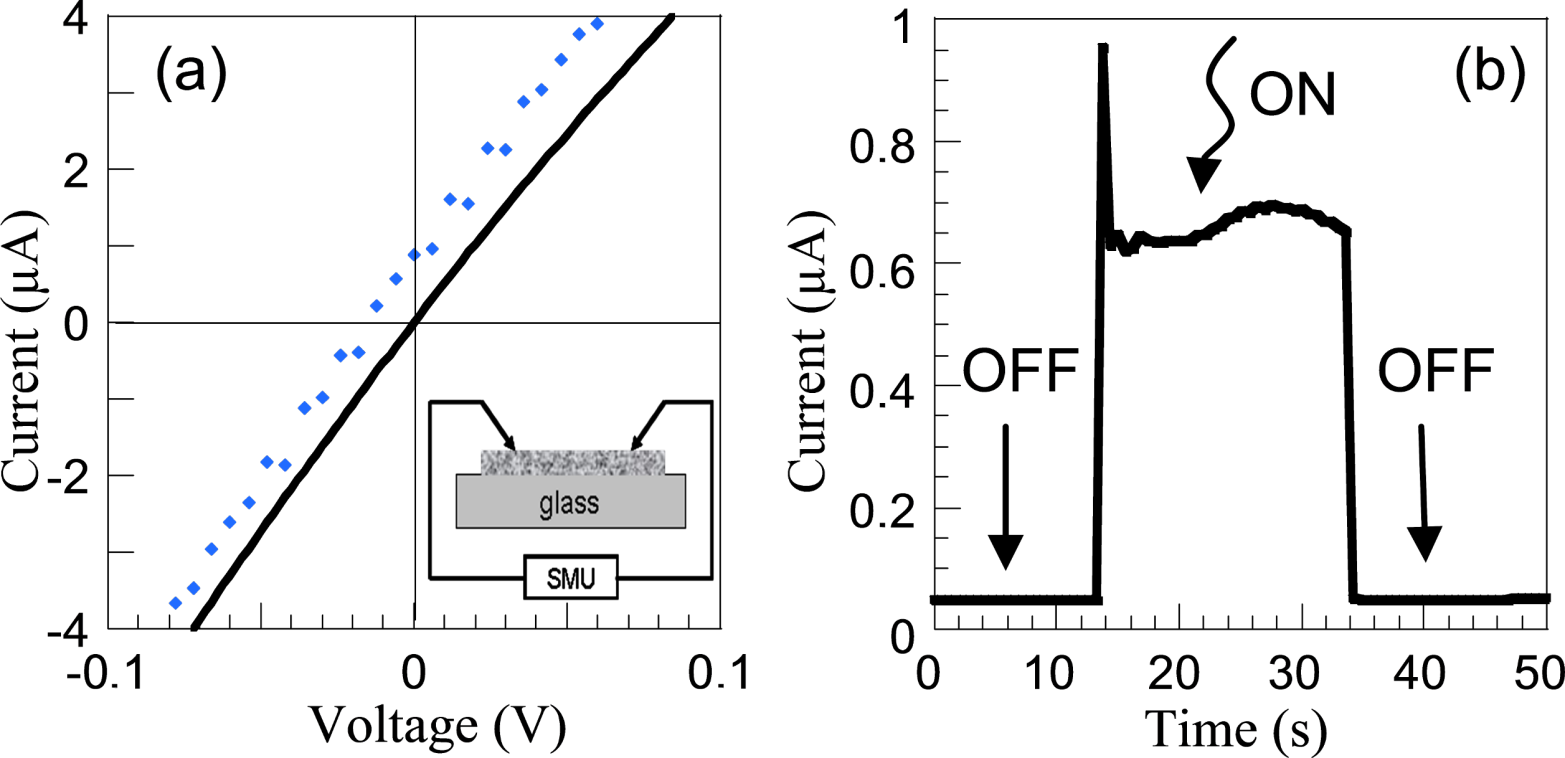
(a) *I*–*V* characteristic of a nanohybrid material in the dark (full line) and under illumination (dots) vs bias voltage. In the inset, a schematic illustration of the measurement set up is given. (b) On/off cycle photoresponse of MWCNT:HfO_2_ excited with UV–vis light (at 10 mV).

For the electrical characterization, the nanocomposite is deposited on a glass slide and contacted using a micromanipulator manual probe station as shown in inset of Figure 5a. The dark current–voltage characteristic is mostly linear, as expected for an ohmic conduction through metallic MWCNTs (Figure 5a). No influence of the concentration of HfO_2_ NPs decorating the MWCNTs is noticed in the dark *I*–*V* curves. UV illumination (365 nm) also has no detectable effect on bare MWCNTs. Although it was reported that MWCNTs generate a photocurrent [62], this effect is generally weak with respect to the dark current in the absence of additional Schottky or p–n junction to enhance the photoresponse [63]. This agrees well with the PL measurements of the nanocomposites (see Figure 2) showing no additional spectral features apart from emission peaks associated with Hf^3+^ and O^2−^ states in HfO_2_ and thus supporting the metallic nature of the MWCNTs. For the MWCNT:HfO_2_ hybrid material, a clear shift of the *I*–*V* curve is observed in Figure 5a upon UV illumination. In the present experiment, the short-circuit current (*I*_SC_) is 0.7 µA and the open-circuit voltage (*V*_oc_) is −12 mV. The photoresponse under UV excitation is sizeable with a higher quantity of the agglomerated HfO_2_:CNT density. The *I*–*V* characteristic is still mainly linear, with a relatively weak decrease of the resistivity, which is consistent with the fact that the electrical transport is dominated by the conduction through the metallic MWCNTs [64]. Under zero bias a photocurrent is generated, indicating that the MWCNT:HfO_2_ nanocomposite acts as a photovoltaic cell. The on/off cycle measurements show a square and well-defined photoresponse after turn-on and turn-off illumination (Figure 5b).

After turn-on illumination, the photocurrent first presents a rapid response time defined by an initial spiking of the photocurrent, indicating a rapid filling and discharging of the defects states. This is followed by a rather stable and reproducible photoresponse, suggesting that the photogenerated electrons are effectively transferred from the NP towards the CNT. Correspondingly, when the light is turned off, the photogenerated charges are rapidly dissipated. Similar to MWCNT:ZnO [64–65], the rapid response of the photocurrent could be related to the intimate contact between the HfO_2_ NP and the MWCNT further enhanced by the absence of functional groups on the surface of the CNT.

In our previous work, we reported on the time evolution of the PL response from the HfO_2_ nanoparticles [21]. At this point, it is worth noting that the time evolution of the luminescence from the cubic HfO_2_ nanoparticles under constant UV exposure (325 nm) shows a steady decline during the first 60 s and the most prominent decrease occurs for emission at 3.1 eV, which is related to Hf^3+^ defects via oxygen vacancies [19]. These defects are located mainly close to the surface and thus dominate the PL due to the large surface-to-volume ratio of the nanoparticles. This also suggests that particular band bending and charge state conditions at the surface define the radiative or nonradiative behavior of the defect center and in turn determine both the spectral features and photoresponse of the hybrid material. Apparently, these surface defects act as radiative centers upon contacting HfO_2_ NPs with CNTs (Figure 3a) and also produce a continuous, quantifiable photocurrent during the on cycle. The abrupt decrease to zero photocurrent during light-off conditions confirms that the remaining trapping centers and nonradiative recombination sites are rapidly dissipated.

These preliminary results demonstrate the potential of the HfO_2_ NP/MWCNT nanocomposite for functional interactions in energy harvesting applications. Quantum efficiency measurements are under investigation in order to precisely evaluate the origin of the photocurrent. Moreover, in order to optimize the structure, it would be necessary to further understand the role of surface defects, notably Hf^3+^ and oxygen vacancies during the photoemission process. In particular, the role of the defects should be evaluated because charge accumulation within them during illumination may induce band bending in the electronic structure, amenable to photocurrent generation.

## Conclusion

We have successfully synthesized a hybrid material composed of nonfunctionalized CNTs and HfO_2_ nanoparticles capable of generating a photocurrent under UV excitation. STEM studies have shown us that the HfO_2_ nanoparticles remain undamaged upon sonication and are preferentially attached to defects in the CNT, such as bends, kinks and buckles. Furthermore, the nanoparticles also tend to anchor on side walls of the CNTs, where amorphous carbon or wall damage is present. Defect sites and amorphous carbon contain a large number of C dangling bonds that act as anchoring sites. The origin of this amorphous carbon is certainly the result of wall damage or C–C bond breaking due to the sonication treatment. Optical measurements have revealed unique features of the hybrid material. In fact, the π-plasmon peak of the CNT gradually overlaps with the band gap absorption edge of the HfO_2_ nanoparticles. This implies that a possible antenna effect of the CNTs on HfO_2_ is likely if the material is excited in the deep UV region. The photocurrent measurements further indicate that evacuation of charges from the surface states of HfO_2_ nanoparticles via direct contact with MWCNTs turns them from radiative to nonradiative recombination centers and contributes to photocurrent generation in the material. Such a material therefore finds interest for applications in photovoltaics owing to an increase in its spectral range along with the plasmonic effect of the CNTs, which also serves to conduct charges from the surface states of the nanoparticles to an external load. To put the present work into perspective, we note that fabrication of all-carbon nanocomposites by replacing HfO_2_ by carbon quantum dots (CQDs) might appear as an appealing continuation of this study considering the very attractive properties of CQDs (e.g., stability, high conductivity, strong and tunable photoluminescence emission throughout the visible spectrum) [66]. However, recent reports show that the quenching in such a system is static and no electron transfer is available for the generation of a photocurrent [11].

## Experimental

Synthesis: The procedure for synthesizing cubic HfO_2_ NPs was carried out in a glove box (O_2_ and H_2_O, <1 ppm). In a typical synthesis, hafnium *tert*-butoxide ((Hf(O*t*-Bu)_4_) precursor (STREM 99.9%) (0.87 mmol) was added to 20 mL (183 mmol) of benzylamine (purified by redistillation (99.5%), Aldrich). The reaction mixture was transferred into a stainless steel autoclave and carefully sealed. Thereafter, the autoclave was taken out of the glove box and heated in a furnace at 300 °C for 2 days. The resulting milky suspensions were centrifuged; the precipitates were thoroughly washed with ethanol and dichloromethane and subsequently dried in air at 60 °C [21]. NANOCYL NC7000 MWCNTs with an average diameter and length of 10 nm and 1.5 μm, respectively, were used in the synthesis. The nanoparticles were then dispersed in pure ethanol along with the MWCNTs and sonicated for a total of 2 h [67].

Characterization: High angle annular dark field scanning transmission electron microscopy (HAADF-STEM) was carried out on a probe-corrected Titan G2 80–200 kV operating at 80 kV to reduce beam damage. The probe size and therefore the point-to-point resolution was ≈1 Å in STEM mode. High resolution transmission electron microscopy (HRTEM) carried out on the same microscope at 200 kV provided a point-to-point resolution of 2.4 Å. Electron energy loss spectroscopy (EELS) was acquired in STEM mode with an Enfinium spectrometer at 80 kV. For the EELS data acquisition, the convergence and collection angles were set to 14.6 mrad and 24 mrad, respectively, for a camera length of 4 cm, a condenser aperture of 50 µm and a spectrometer slit of 3 mm. For these values, the energy resolution measured as the full width at half maximum of the zero-loss peak is 0.9 eV and the dispersion was set to 0.4 eV/channel.

The absorption properties were derived from the transmittance measurements performed at room temperature using a UV–vis spectrophotometer (Thermo Scientific, EVO-600). PL was investigated at a room temperature by employing a 325 nm wavelength CW He–Cd laser with an output power of 6 mW as an excitation source. The emission was collected by a microscope and directed to a fiber optic spectrometer (Ocean Optics, USB4000) with a spectral resolution of 2 nm. The electrical measurements were carried out using a source measure unit (Agilent 4156). The sample was illuminated by a 125 W Hg lamp emanating a wavelength of 365 nm.
